# Evaluating harm associated with anti-malarial drugs: a survey of methods used by clinical researchers to elicit, assess and record participant-reported adverse events and related data

**DOI:** 10.1186/1475-2875-12-325

**Published:** 2013-09-16

**Authors:** Elizabeth N Allen, Clare IR Chandler, Nyaradzo Mandimika, Cheryl Pace, Ushma Mehta, Karen I Barnes

**Affiliations:** 1 Division of Clinical Pharmacology, Department of Medicine, University of Cape Town, Cape Town, South Africa; 2 Department of Global Health & Development, London School of Hygiene & Tropical Medicine, London, UK; 3 Department of Clinical Sciences, Liverpool School of Tropical Medicine, Liverpool, UK

**Keywords:** Anti-malarial, Malaria, Harm, Adverse event, Concomitant medication, Adherence, Assessment, Safety, Method, Harmonize

## Abstract

**Background:**

Participant reports of medical histories, adverse events (AE) and non-study drugs are integral to evaluating harm in clinical research. However, interpreting or synthesizing results is complicated if studies use different methods for ascertaining and assessing these data. To explore how these data are obtained in malaria drug studies, a descriptive online survey of clinical researchers was conducted during 2012 and 2013.

**Methods:**

The survey was advertised through e-mails, collaborators and at conferences. Questions aimed to capture the detail, rationale and application of methods used to obtain relevant data within various study designs and populations. Closed responses were analysed using proportions, open responses through identifying repeating ideas and underlying concepts.

**Results:**

Of fifty-two respondents from 25 counties, 87% worked at an investigational site and 75% reported about an interventional study. Studies employed a range of methods to elicit, assess and record participant-reported AEs and related data. Questioning about AEs in 31% of interventional studies was a combination of general (open questions about health) and structured (reference to specific health-related items), 26% used structured only and 18% general only. No observational studies used general questioning alone. A minority incorporated pictorial tools. Rationales for the questioning approach included: standardization of assessment or data capture, specificity or comprehensiveness of data sought, avoidance of suggestion, feasibility, and understanding participants’ perceptions. Most respondents considered the approach they reported was optimal, though several reconsidered this. Four AE grading, and three causality assessment approaches were reported. Combining general and structured questions about non-study drug use were considered useful for revealing and identifying specific medicines, while pictures could enhance reports, particularly in areas of low literacy.

**Conclusions:**

It is critical to evaluate the safety of anti-malarial drugs being deployed in large, diverse populations. Many studies would be suitable for contributing to a larger body of evidence for answering questions on harm. However this survey showed that various methods are used to obtain relevant data, which could influence study results. As the best practices for obtaining such data are unclear, anti-malarial clinical researchers should work towards consensus about the selection and/or design of optimal methods.

## Background

When investigating drug effects, study results are influenced by the methods used to collect, assess, record and report outcomes. Studies using different methods can be difficult to evaluate within a body of evidence, so it is beneficial to harmonize conduct [1]. There has been significant harmonization of methods relating to efficacy outcomes. For malaria, this entails a World Health Organization (WHO) protocol for therapeutic efficacy evaluations and a categorization of treatment response developed by the WorldWide Antimalarial Resistance Network (WWARN) [2,3]. In general there has been less attention to developing harmonized methods for evaluating safety (monitoring the presence or absence of harm), reflecting its historical marginalization within drug development [4-6]. Notable exceptions include case definitions for adverse vaccine reactions, standard assessment and reporting of adverse effects in rheumatology, HIV and oncology, and instruments for determining specific anticipated effects, for example, extrapyramidal motor side effects of antipsychotics [7-11]. Little work concerns the systematic detection of unanticipated effects [12]. For malaria trials, WHO recommends that, to ascertain the incidence of adverse events (AEs), participants be asked about symptoms that have emerged since the previous follow-up visit by “direct questioning” [2].

The manner of ascertaining AEs from subjective participant reports may be more problematic for harmonization compared to those determined through tests or examinations, as it is proposed the former are shaped by memory, expectations, consideration of information required and willingness to report [13-15]. The interpretation and comparison of AE data is further complicated if studies use different methods for assessing severity and relationship to study drug. Research shows that questioning (elicitation) methods can play a role; when asked about specific conditions or body systems as opposed to general questions about health, participants typically report more [16]. This suggests certain techniques overcome particular barriers to reporting [13]. Some, therefore, recommend using questionnaires or checklists of potential AEs due to their greater sensitivity [17]. However, opinion is divided; despite evidence that participants do not always report an AE when asked a general question, some counsel against more detailed questioning so as to prevent inducing a particular response [9,18]. Others suggest that AEs detected by detailed questioning are not as clinically meaningful as those mentioned spontaneously and that general questioning provides a better evaluation of drug-placebo difference [19,20]. That this initial stage of collecting AE data has been largely excluded from efforts to harmonize safety evaluation methods within some therapeutic areas perhaps reflects these complexities and debates.

Previous medical histories, non-study drugs (previous or concomitant medication), and study drug adherence data, all largely ascertained from participant reports, are integral to assessing harm. Yet these are rarely included in debate about the challenges of obtaining adverse effect data, despite evidence that participants fail to report some medications when asked [21]. As for AEs, the questioning tool has been found to influence reports [15,22].

It has been acknowledged that the above factors inherent in evaluating anti-malarial drug safety and tolerability are challenging, and that there is a need for further guidance [23]. To contribute to debate about these concerns a survey was conducted about the methods researchers use to obtain data for evaluating participant-reported harms. A survey was chosen over a literature review as the detail of how data are collected and managed is insufficient in most publications [24]. A survey could also prepare for subsequent collaborative work within the anti-malarial research community, with the aim of selecting or designing suitable harmonized methods. This survey was conducted within the ACT Consortium, a group of researchers conducting projects relating to the wide-scale implementation of artemisinin-based combination therapy [25]. Several projects contribute safety outcome data to a register coordinated by the Liverpool School of Tropical Medicine (in collaboration with the Malaria in Pregnancy, MiP, Consortium), which can produce individual study reports and pooled analyses, and can also accept data from outside of the ACT Consortium [26]. This will be a valuable resource for understanding more about the harmful effects of anti-malarials, particularly as drugs are being widely distributed within high-risk populations who could be quite different to those studied within registration clinical trials.

## Methods

### Survey objective

The objective was to explore the methods used to detect, assess and record participant-reported medical history, AE, study drug adherence and non-study drug data in anti-malarial clinical drug research.

### Survey population and sampling

Those eligible were anyone involved in the elicitation (by questioning) and recording of harms and related data from participants in any malaria clinical drug study, whether directly interacting with participants, or designing questioning, assessment or data recording methods, or taking responsibility for these tasks. There was no sample size calculation due to the nature of this descriptive survey. Instead, the survey was as inclusive of respondents as possible within the available budget and timelines. Recruitment was active and passive. Personal e-mail invites were sent to known contacts and potential eligible respondents identified from PubMed, clinicaltrials.gov, the Pan-African Clinical Trial Registry and the Initiative to Strengthen Health Research Capacity in Africa’s database of African Health Researchers [27-30]. The ACT Consortium, MiP Consortium, WWARN, and Global Health Trials publicized the survey through newsletters, webpages or mailing lists, and it was advertised at several conferences [25,26,31,32]. During recruitment it was determined that sponsors may not always know the detail required, though would be valuable in subsequent debates, so they were not actively targeted further. Survey respondents were asked to coordinate responses within project teams unless members had differing experiences. Self-selection ultimately determined who participated.

### Survey conduct and analysis

The survey (Additional file 1) was developed in SurveyGizmo® for completion online from August 2012 to January 2013 [33]. Questions captured the detail, rationale and application of methods used within various malaria drug study designs and populations. Respondents were also asked about important and feasible approaches over and above what they had experienced. Examples of tools (eg, case record forms) and recommended literature were requested. It was too complex to ask about trial design, such as length of participant follow-up, although these are important aspects of assessing AEs that can also hinder meta-analyses [24]. The questionnaire was piloted with seven eligible respondents, establishing whether questions were understood as intended, and requesting suggestions for improving content and conduct. That the survey was considered acceptable and relevant contributed to face and content validity. The survey was anticipated to take 20 minutes to complete. After recruitment, content was downloaded into Microsoft Excel®. Closed question responses are presented using proportions, and open responses, through mapping underlying concepts and repeating ideas identified [34].

### Ethical considerations

The University of Cape Town’s Faculty of Health Sciences Research Ethics Committee gave written approval for the survey, and consent to take part was integral with its completion. Access to responses was restricted to those in the investigational team. Once analysis was complete the survey was de-activated, removing links between e-mail addresses and the website.

## Results

### Survey respondents and nature of studies described

**Figure 1:**
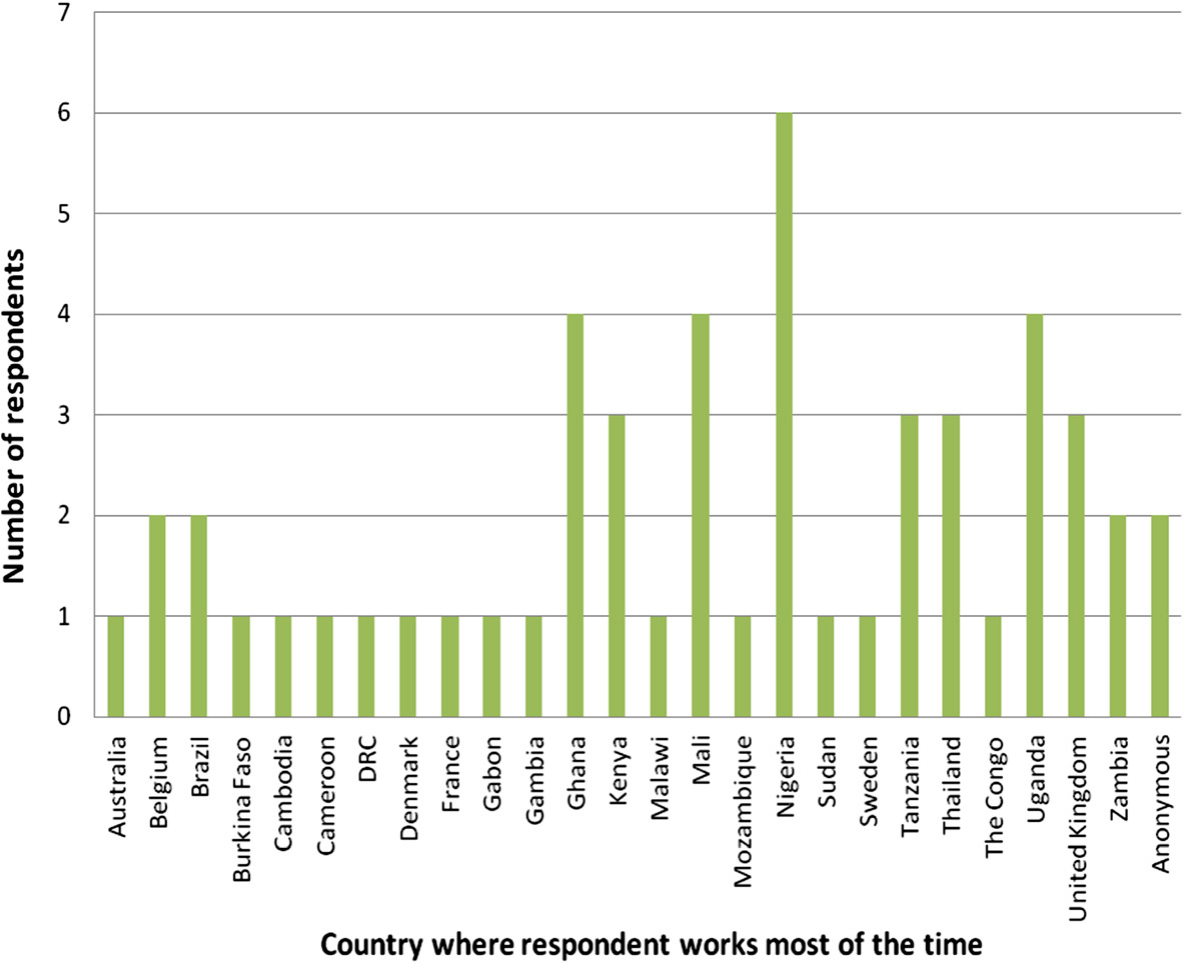
Survey respondents by geographical region where respondent works most of the time (N = 52).

There were 150 non-duplicate visits to the survey and 56 questionnaires sufficiently completed for inclusion. Four were excluded as they concerned vaccine trials which were not the focus of the survey. Of the remaining 52, there was representation from 25 countries (Figure 1). Eighty-one per cent of respondents had more than five years’ involvement in malaria clinical research. Most (85%) worked at a study site; in addition there were four representing sponsors/sponsor-investigators, four taking other coordinating roles and an advisory board member. Twenty-five (57%) site respondents were Principal Investigators (PIs), the remainder being co-investigators/researchers (n = 10), study coordinators (n = 7), or other/unknown (n = 2). Forty-three (84%) took responsibility for selecting or developing the methods used to collect participant-reported AE and/or non-study drug data. The majority of clinical research conducted by respondents was non-commercial (77%).

**Figure 2:**
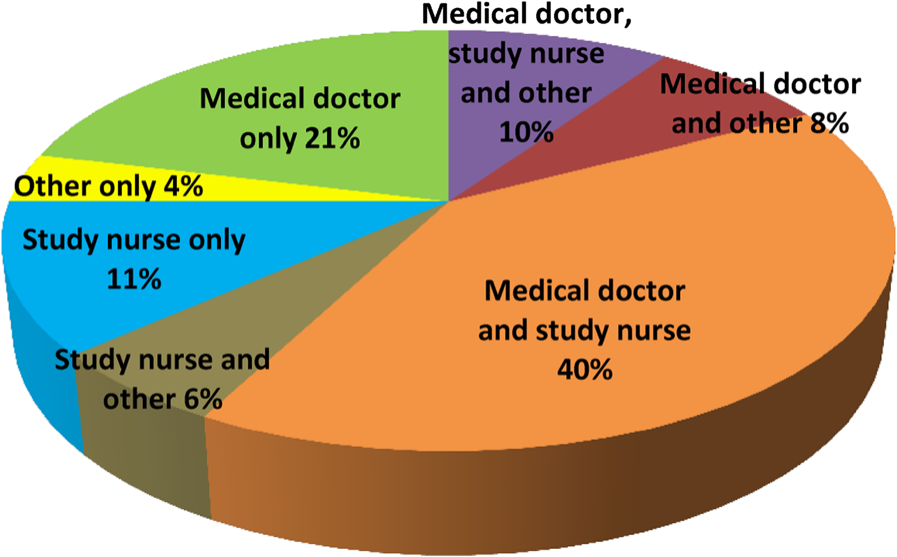
**Proportion of studies by staff member(s) who question participants about health and drug use (N = 52).** “Other” most frequently referred to community health workers, field workers and various trained members of the research team.

Respondents answered the survey with reference to their most recent study, of which 39 (75%) were interventional, nine (17%) observational, the remainder undefined. Studies employed a variety of staff to take a medical history from participants or care-givers, including any change in health or use of treatments (Figure 2). Sixty per cent of studies included children between one and 17 years old; the median age in studies where children were asked directly about their health was five years, while for medication-use it was seven years. Forty per cent (20/50) involved a translator in participant conversations. The limited number (n = 5) and diversity of the AE data collection tools submitted meant that they could not be explored usefully. Hereafter N = 52 unless otherwise indicated (not all questions were relevant for, or answered by, all respondents).

### Questioning study participants about health to collect AE data

**Figure 3:**
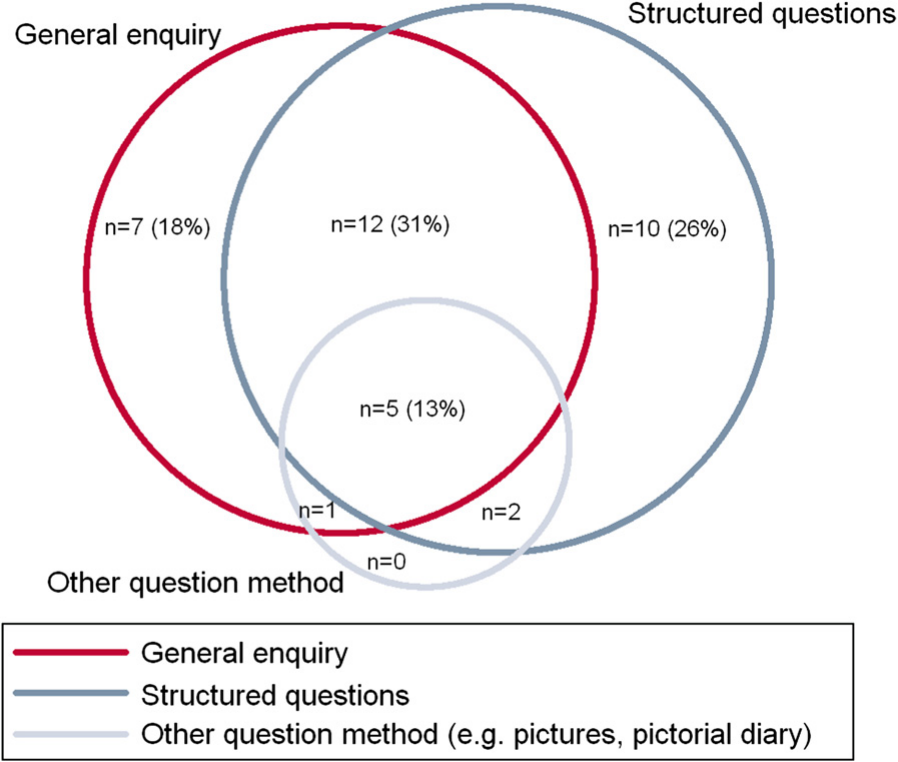
Questioning methods about health used to capture AE data in interventional studies (N = 39, 2 = not known).

A range of methods were applied to elicit participant-reported AEs. Questioning in 12/39 (31%) of interventional studies was a combination of general (without reference to particular conditions or body system) and structured (with reference to particular conditions or body system), while 10/39 (26%) were structured only and 7/39 (18%) general only (Figure 3). All of the nine observational studies described incorporated structured questioning. General enquiries involved phraseology about one of the following concepts (in descending order of frequency by two or more respondents): general enquiry about feeling (eg, *“How have you [has your child] been feeling?”*), explicit enquiry about change in health (eg, “*Have you observed any change or new complaint*”), and enquiry with implied causality reference (eg, “*Did your child experience any serious side effect from the drug*”). One respondent used a phrase possibly aimed at overcoming some impediments to reporting: *“Can you confide in us to give a sincere answer”*. When general phrases were mandatory for use in a study, all respondents were confident the phrase was used as prescribed (N = 12).

Structured enquiries involved numerous permutations of symptoms (malaria-specific or not), body systems, and expected adverse drug effects. When used, they were mostly in reference to a prepared list (85%), either exactly as prescribed, paraphrased or a combination. Other than general and structured questioning, eight studies (N = 52, 14%), 5 of which were interventional (N = 39, 13%) incorporated other methods, including pictures and/or pictorial diaries (eg, clinical presentations). Only one such tool was referenced [35].

Key attributes valued in the rationale for method choice were: *standardization* of AE assessment or data capture, *specificity* or *comprehensiveness* of data sought, *avoidance of suggestion* of a certain response, *feasibility* and *understanding participants’ perceptions about health* (Table 1). Other than more structured enquiries being used to obtain comprehensive reports, there was overlap in the use of different questioning methods to fulfil the same rationales. Most respondents (35/47, 74%) said the approach used in their last anti-malarial study to elicit participant-reported AEs was the one they also considered optimal (important and feasible). Two of these reported that the original method worked or was reliable, though no details were given on validity or reliability assessments. Five respondents said, however, that the method depended on the needs and circumstances of the study or participant population, and 7 suggested an alternative to their originally reported method; one moving from a combination of general and structured questioning to only general questioning (to reduce bias in reporting) and six by proposing a more thorough strategy, incorporating further types of questioning or tools. For the latter, pictures, wall charts, and translations of instruments into local languages were deemed potentially useful for obtaining comprehensive data, and beneficial in areas of low literacy. Seventy-seven per cent (36/50) used the same method of assessing health at baseline as for follow-up visits to detect AEs. Reasons for using a different approach at baseline were to target malaria symptoms and eligibility criteria, including drug-related allergies.

**Table 1:** Rationale for choice of questioning method about health and non-study drug treatment (28/52 respondents)

	**Example quotes from respondents**	**Question method used in the study**			
		**General enquiry only**	**Structured enquiry only**	**General and structured enquiry combination**	**Added pictures, diary, charts, collecting packets, showing sample drugs**
**Standardization of assessments or data capture** (including historical use of a method in the research group)	*“A systematic approach based on pharmacovigilance procedures developed by our collaborators”*	x	x	x	
	*“We are used to that”*				
**Specificity of data sought** (seeking information about particular adverse events, malaria symptoms or drugs)	*“We wanted to find out about specific symptoms and adverse effects”*	x	x	x	
	*“The named drug questions targeted drugs of special interest”*				
**Comprehensiveness of data sought** (participant guidance, report clarification, overcoming barriers to reporting such as poor recall or ability to name medicines)	*“To provide a clear understanding about what investigators are looking for and to be sure they capture all complaints from study participants”*		x	x	x
	*“To get more information which may have been missed during the initial interview”*				
**Avoidance of suggestion**	*“Keeping questions open and not leading so that only events significant to the patient are reported”*	x		x	
**Feasibility**	*“A simple screen [as the] main focus of the study was not safety/tolerability”*	x	x	x	
	*“Appears simple and not complex”*				
**Understand participants’ perceptions about health**	*“[To] know if [symptoms] are related to chronic disease or traditional belief”*		x	x	

### Assessing and recording AE data

A greater proportion of studies in which AEs were not assessed for severity were observational (63 *versus* 11% of interventional study reports). Of 20 respondents describing an AE severity assessment method (19 interventional and one observational), 30% used a tool published by the WHO, 20% by the Division of Microbiology and Infectious Diseases, and 10% by the US National Institute for Cancer [7,8,36]. One respondent commented that the WHO tool does not work well for neutropenia and other variables in their setting, citing Saathoff et al [37]. The remaining 40% (all interventional), used the categories “mild, moderate, severe” (or synonyms), with various definitions largely based on the concept of the AEs’ impact on daily living. Methods used to determine the relationship between AEs and the study drug(s), included the WHO-Uppsala Monitoring Centre causality tool, Bradford Hill criteria, and a range of in-house categories or those adopted from unknown sources [38,39]. Forty per cent (19/47) said study participant AE reports were entered verbatim into their database, either solely or with the staff member’s or standard terminology for the event. Standard terminologies were largely protocol-specific, though three interventional study respondents cited use of MedDRA® [40].

### Non-study drug use

There were no obvious differences between observational and interventional studies in how non-study drug data were elicited. The majority described general questions (41/47, 87%), English terms being ‘medicine’, ‘medication’ or (less often) ‘drug’. There was no common practice for whether these phrases were required to be used by study staff or not. Categories explicitly referred to in general enquiries included: prescription-only medicines (32/39, 82%), over-the-counter medicines (27/39, 69%), traditional treatments (24/39, 62%), supplements (11/38, 28%), and vaccinations (8/39, 21%). Sixty per cent (28/47) of respondents used a structured medication enquiry, largely in combination with the general enquiry. About half of these incorporated a study-specific tool specifying treatment class and/or name. There were two reports of using pictures of either a mosquito or drug packets to elicit non-study anti-malarial drug use.

Structured enquires were deemed useful for revealing specific medicines, particularly anti-malarials, and to help name medicines mentioned in response to a preliminary general enquiry. Pictures or diaries were considered to enhance reports and were useful in areas of low literacy (Table 1). Sixty-six per cent (27/41) of respondents were comfortable the approach they used was optimal. Those indicating it could be improved, suggested augmenting a general enquiry with specific mention (or pictures) of medicines such as anti-malarials or drugs potentially interacting with the study drug. One person recommended the content of such lists be developed from pre-study qualitative work in the target population, though another described pictorial tools as “*good but logistically hard and…costly to develop”.* A further proposal was for all staff in contact with a participant to enquire about non-study drug use. There was mention of inherent limitations to questioning about non-study drug intake, “*To get medical attention [study participants] lie they have not taken anything*”, and one respondent suggested assaying blood drug concentrations.

### Study drug adherence data

Eighty per cent of reported studies (33/41) collected study drug adherence data using combinations of dosing being directly observed wholly or partially (n = 24), participant recall (n = 14), pill counts (n = 7), dispensing confirmation (n = 8), and pill diaries (n = 2). In 65% or more of responses (N = 32), data were captured regarding quantity of doses dispensed, doses observed, duration of total therapy, time of doses and whether the participant vomited. About half of respondents reported capturing the reason for non-adherence and whether the study drug was taken with food (where applicable). When patients vomited the usual practice was to repeat the dose within a specified period and make a note in the records. Definitions for adherence included taking all doses appropriately, the proportion of participants who took at least a specified percentage of study drug (80 or 90%), and use of the categories adherent/probably non-adherent/definitely non adherent. Seventy-nine per cent said that adherence was described in the study report.

## Discussion

Exploring processes for evaluating harm in anti-malarial drug research may offer insight into how study participants’ experiences become facts in databases. This was the first investigation into the detail of methods used within malaria clinical drug research to elicit, assess and record participant-reported harms and associated data. These are critical components of anti-malarial drug safety evaluations, including systematic reviews and meta-analyses, yet are not readily available in publications [41]. Fortunately, this situation is generally improving [42]. An online survey was pragmatic and had participation from 25 countries, representing over 50 studies.

### Elicitation of AEs

The survey showed that various permutations of general and structured questioning are used to elicit participant-reported anti-malarial AEs, with a minority of studies incorporating pictorial tools. The concern is that differences in questioning methods, even nuances between general enquiries, influence data elicited [16,18]. The few case record forms submitted validated the survey findings that specific general enquiry phrases are obligatory in some studies. While conversations are unlikely to follow a script completely, a preliminary standard phrase can orientate staff and participants to the intention of questioning. Structured questioning for AEs with reference to predefined fields was frequently used to permit standardized assessments and data capture, and obtain comprehensive replies. While structured enquiries can increase the number of AEs reported (hence burden on the study team) they can also facilitate a tolerability assessment, which is particularly important at a population level [23]. The survey results reflect the literature that children from five to seven years old can contribute valuably in medical consultations. As young children may be marginalized in conversations, however, it is proposed that study staff are guided in managing triadic communications [43].

Most respondents considered the questioning approach they used was optimal. However, the subtle shifts observed between rationale for methods reported in use and respondents’ perceptions of optimal approaches indicate reflection on appropriate methods, and particularly the potential value of novel methods. In other therapeutic areas, work is underway to harness techniques used in developing validated patient-reported outcome measures (PROs) for AEs, a trend being self-report via the internet [44,45]. Many existing instruments, however, restrict the development of items (i. e. potential AEs) to those side-effects already associated with a drug by patients and/or experts, and generate summed scores rather than individual AE reports [46]. This may not allow for adequate detection of unexpected AEs or facilitate a traditional safety evaluation through incidence measures. Nevertheless, finding ways to better represent a participant’s experience of an AE is important. For malaria it makes sense to pursue methods suitable for areas of lower literacy, including pictures, mobile phones, or innovative methods being explored in other areas of health care communication, such as when patients indicate health problems on body outlines kept at home for later discussion at the clinic [35,47].

The survey results did not substantiate whether variation in AE enquiry methodology is a factor of study design. This issue should be debated; for instance, whether a more sensitive enquiry is used to detect AEs in earlier phase trials, compared to later phase studies where a drug has a better understood safety profile. The caveat, however, is uncertainty about which questioning method(s) produce valid data and whether there should be consistency throughout study visits; while it is important to avoid leading patients to a particular response, it may also be inappropriate to use a less sensitive measure to detect change in health post-intervention than one used to determine a baseline. There should be further methodological research to investigate these concerns, including qualitative work in both participants and staff about understanding of terminology and meaningful outcomes in malaria-endemic populations. The ACT Consortium plans to contribute through a Cochrane review of research comparing adverse effect elicitation methods [48]. Available data could potentially be distilled to relevant concepts for local adaptation according to the study design and population.

### Grading severity of AEs

A variety of AE severity/toxicity grading tools and methods for determining relationship with study drug were reported. Researchers in other therapeutic areas regularly debate the development of disease-specific toxicity criteria for end points important to health practitioners and patients [9]. Should malaria researchers do the same, such criteria should be based on locally relevant reference ranges for laboratory parameters [49]. Staedke *et al* observed variability between sites in assessing AEs for severity and causality when using standard grading scales, reflecting differing value judgements of staff and participants [23]. There is a need then to think carefully about terminology and application of criteria so that there is consistent interpretation [50]. Similarly, because of the plethora of causality assessment criteria used in clinical research, there have been recommendations of simpler categories (eg, related/not-related; reasonable possibility/no possibility of attribution), with more emphasis on determining causality by statistical methods where possible [18,50].

### Eliciting and recording study and non-study drug data

Survey respondents considered combining structured and general questions about non-study drug use useful for revealing and identifying specific medicines. Pictures, diaries and samples etc, were seen to help with recall and participant understanding of what to report, particularly pertinent for areas of low literacy. The cost of formative work for development of such tools may be prohibitive for individual study teams, but could be shared with other groups recruiting from a similar patient population (even if studying different therapeutic areas). Often daily anti-malarial doses can be fully observed. However, when required (e g, for twice daily dosing of artemether-lumefantrine), there were various methods reported as being used for measuring adherence, whether indirect and objective (pill counts and dispensing information) or indirect and subjective (diaries and recall). Combining recall questionnaires or pill diaries with an electronic pillbox and count may provide the most accurate information, though in areas of low literacy or limited study budget a recall questionnaire with pill count may be more realistic (WWARN, personal communication, 2012). More work is needed to define adherence for the variety of drugs or combinations used in malaria to inform meta- or pooled data analyses.

### Limitations

Surveys inherently have constraints in terms of their reach, respondents’ understanding of questions and their ability or willingness to give comprehensive answers. It is assumed that responses reflect the methods used in practice. However, it would have strengthened the study if other field staff were included to know whether their responses differed from those of the PIs, co-investigators, study coordinators and researchers who took part. The survey results do not represent all anti-malarial drug clinical research studies and it was not possible to detect differences in methodologies between study designs or populations. It is hoped that any such limitations will be overcome in future debates. In particular, more representation from sponsor organizations and regulatory authorities will be key.

## Conclusion

Many anti-malarial drug studies would be suitable for contributing to a larger body of evidence for answering questions on harm, regardless of design or whether safety was the primary objective [51,52]. Meta-analysing and pooling data can increase the power to generate new signals, and clarify the incidence and possibly drivers of known harms. Applying these tools is increasingly urgent as large populations, some who may have multiple co-morbidities or be taking concomitant medications, are exposed to anti-malarial drugs. Should anti-malarials be distributed in asymptomatic or malaria negative populations it is particularly important to evaluate benefits versus risk [53]. Studies evaluating strategies such as intermittent presumptive treatment, screening and treatment, and mass drug administrations should assess safety, including the less severe adverse effects as tolerability can impact on community acceptance of, and individual adherence to medicines [54]. In order to synthesize study results effectively researchers need to move towards harmonized methods for obtaining safety data. As there are many unanswered questions about the best practices for eliciting, assessing and recording such data, priority areas for further work should be established through dialogue within the anti-malarial clinical research community. This could include 1) nesting methodological research within studies to find optimal approaches or tools for questioning participants to obtain AEs and related data, 2) deciding whether to adopt an existing toxicity grading scheme from another therapeutic area or develop one for malaria endemic populations, or 3) developing guidance on use of a common causality assessment tool. User-friendly open access databases suitable for a range of study designs could also be developed collaboratively to help researchers manage their data efficiently [55]. Where appropriate these should incorporate harmonized fields and terminologies so that they may be used more widely in the non-commercial sector. The ACT Consortium plans to contribute by facilitating a Delphi process; a summary of the available literature will be presented to interested malaria clinical researchers who will then work towards consensus about the appropriate design of relevant and feasible methods to detect these important data. This work could also catalyze much needed progress in the detection and investigation of harms in other therapeutic areas, generally improving the ability to compare or synthesize studies [56].

## Competing interests

All authors receive full or partial salary support through the ACT Consortium or partial support through WWARN.

## Authors’ contributions

EA conceived of the survey, led design, data collection and analysis, and wrote the paper. CC, UM, CP and KB had input throughout while NM conducted data management and contributed to data analysis. All authors approved the final paper.

## Supplementary Material

Additional file 1 **Survey questionnaire.** Content of online survey. Click here for file
