# Supplementary material for: Evaluating harm associated with anti-malarial drugs: a survey of methods used by clinical researchers to elicit, assess and record participant-reported adverse events and related data
Source: Malar J. 2013 Sep 16;12:325. doi: 10.1186/1475-2875-12-325 (PMC3848530; doi:10.1186/1475-2875-12-325)
Supplement: Additional file 1 — Survey questionnaire. Content of online survey. [file 1475-2875-12-325-S1.pdf]

Survey questionnaire

Welcome

Elicitation and Recording of Participant-reported Safety Data in Malaria Clinical Drug Trials/Studies

We are interested in talking to malaria clinical research team members about the details of how malaria clinical research participants (or their caregivers, such as a parent) are asked about their health and treatment-taking in order to collect medical history, adverse event and concomitant medication (safety) data. The survey is not about spontaneous reporting of adverse drug reactions (yellow-card, prescription event monitoring etc.) or vaccine clinical research.

This voluntary survey should take about 15-20 minutes. We would like to be able to follow up with you about specific methods you have used and have the opportunity to work with you in future collaborations. We would therefore be grateful if you would provide your name and contact details. These will be stored in secure electronic and paper files and will not be used in any report.

Should other members of your research team have used the same methods to collect safety data then one person may respond on behalf of the team. Ideally, this should be a clinical investigator or someone who has been involved in the selection or development of methods used to collect participant-reported safety data.

1) In which country do you work *most of the time*? ( ) Abkhazia to ( ) Zimbabwe

2) Your name: ____________

3) What is the name of the organization where you work? ____________

4) Phone number (please include country and area codes!): ____________

5) Email address: ____________

6) How long have you been involved in *malaria* clinical research?*

( ) Have never been involved in malaria clinical research

( ) < 1 year

( ) 1-5 years

( ) > 5 years

7) Indicate your *most recent primary role* within malaria clinical research? Check the one that fits your role best.*

( ) Working at an investigational site

( ) Representative of a sponsor (other than sponsor-investigator)

( ) Other: _________________

Indicate your *most recent primary role* at an investigational site?

( ) Principal investigator

( ) Co-investigator or sub-investigator

( ) Study coordinator

( ) Research nurse

( ) Research team member other (briefly describe role): _________________

( ) Other: _________________

8) Is the majority of your malaria clinical research sponsored by:

( ) Pharmaceutical companies

( ) Non-commercial entities (e.g. Medicines for Malaria Venture)

( ) Your own institution (i.e. a member of the team is a Sponsor-investigator)

( ) Other: _________________

9) Have you had responsibility for *selecting or developing* methods used to collect adverse event or concomitant medication data in clinical trials/studies?

( ) Yes

( ) No

Please consider your *most recent malaria* clinical drug research study where participants (or their caregiver, such as a parent) were asked about their health and use of treatments to collect medical history, adverse event or concomitant medication data.

10) What type of study was your *most recent malaria* clinical research study? Check all that apply:

[ ] Interventional

[ ] Observational

[ ] Single arm

[ ] Multiple arms

[ ] Randomized allocation

[ ] Other

11) What was the research population of your *most recent malaria* clinical research study? Check all that apply:

[ ] Adults

[ ] Children <1year

[ ] Children 1-5 years

[ ] Children 5-12 years

[ ] Children 12-17 years

[ ] Healthy volunteers

[ ] Patients with malaria

[ ] Other

In general, from about what age were children asked directly about their *health?*

( ) Age in years: _________________

( ) Children were NOT asked directly at all

( ) Don't know

In general, from about what age were children asked directly about their *use of treatments*?

( ) Age in years: _________________

( ) Children were NOT asked directly at all

( ) Don't know

12) Which staff members were involved in asking participants (or their caregivers) about their health and use of treatments? Check all that apply:

[ ] Medical doctor

[ ] Study nurse

[ ] Other

13) Were questions about health and use of treatments ever asked through a translator?

( ) Yes

( ) No

( ) Don't know

<u>Asking participants (or their caregivers) about *health* to collect adverse event (AE) data, at visits *after* the first visit.</u>

14) Were participants (or their caregiver) asked about their *health* since baseline using a general question *without* reference to a particular condition or body system, e.g. 'how have you been feeling?' or 'has your child experienced any problems?'

( ) Yes

( ) No

( ) Don't know

Please give the phrase(s) or question(s) used:

( ) Don't know

( ) Phrase(s) used: _________________

Did the study *require* this particular phrase(s) be used for the general question(s)?

( ) Yes

( ) No

( ) Don't know

How confident are you that all staff used the study-specific phrase(s) *most* of the time?

( ) Confident

( ) Not confident

Asking participants (or their caregivers) about their *health* to collect adverse event (AE) data, at visits *after* the first visit (continued).

15) Were participants (or their caregiver) asked about any *change in their health* since baseline using *structured* questions, e.g. 'please tell me if you have experienced fever, cough, headache….?' or 'has your child had any problems with the chest, head….?'

( ) Yes

( ) No

( ) Don't know

Did the study *require particular* questions be asked, e.g. using a prepared list of possible options?

( ) Yes

( ) No

( ) Don't know

How were health issues defined in the questions? Check all that apply:

[ ] By symptom

[ ] By body system

[ ] Expected adverse events

[ ] Malaria symptoms

[ ] Other

How were options presented? Check all that apply:

[ ] Staff used the exact words as prepared

[ ] Staff rephrased items in their own words

[ ] Items were only presented if they had been reported at a previous visit

[ ] Other

Please *describe* any non study-specific *structured* questions asked:

( ) Question(s) used: _________________

( ) Don't know

16) Were participants (or their caregiver) asked about their *health* since baseline in *another way* to the general or structured method (e.g. picture tool, diary)?

( ) Yes

( ) No

( ) Don't know

Please briefly describe the method(s): ______________

17) What was the rationale for using the above whole approach to questioning about *health* in this study?

18) Was this *same* approach to questioning participants (or their caregivers) about their *health* to collect AEs, also used when asking about medical history at *baseline*?

( ) Yes

( ) No

( ) Don't know

19) How was the questioning about health *different* at baseline? _______________

20) Does the above approach to questioning participants (or their caregivers) about their *health* reflect *most malaria clinical studies* you have been involved in?

( ) Yes

( ) No

( ) Don't know

( ) Other: _________________

Please briefly describe other questioning methods or combinations of methods used: _______________

21) What do you consider as an *optimal* (important and feasible) approach for asking participants (or their caregivers) about their *health* to collect AE data:

( ) The approach reported above

( ) A different approach to my above report (briefly describe): _________________

22) Why do you consider that optimal?

Recording of adverse events (AEs) in the database.

23) How were the participants' (or their caregivers') reports eventually *recorded in the database* as AEs? Check all that apply:

[ ] As verbatim participant (or caregiver) reports

[ ] According to staff members' own terminology for symptom or diagnosis

[ ] Standard terminology (e.g. specific coding method)

[ ] Other

Which standard terminology or coding system was used? _______________

24) How did you assess AEs for *severity*, if at all?

( ) Not assessed for severity

( ) Published grading scale

( ) Other grading scale method

( ) Don't know

Please provide us with a reference to the grading scale or a description of the method used, if known:

25) How did you assess adverse events for *causality*, if at all?

( ) Not assessed for causality

( ) Published causality rating

( ) Other causality rating method

( ) Don't know

Please provide us with a reference to the rating scale or a description of the method used, if known:

<u>**Asking participants (or their caregivers) about their *use of treatments* (other than the study drug) to record previous and concomitant medication data.**</u>

26) Were participants (or their caregiver) questioned about their *use of non-study treatments* using a general question *without* reference to a particular treatment class or name, e.g. 'Please tell me about any treatment you have used' or 'Please tell me about any treatment your child is currently using'?

( ) Yes

( ) No

( ) Don't know

Please give the phrase(s) or question(s) used:

( ) Don't know

( ) Phrase(s) used: _________________

Did the study *require* this particular phrase(s) be used for the general question(s)?

( ) Yes

( ) No

( ) Don't know

Which of the following were explicitly referred to during general questioning (check all that apply):

[ ] Prescription medicines

[ ] Over the counter medicines

[ ] Traditional treatments

[ ] Supplements

[ ] Vaccinations

[ ] Other

27) Were participants (or their caregiver) questioned about their *use of non-study treatments* by asking *specific* or *structured* questions, e.g. 'Have you taken paracetamol, antibiotics….?'

( ) Yes

( ) No

( ) Don't know

Did the study *require particular questions* be asked, e.g. using a prepared list of possible options?

( ) Yes

( ) No

( ) Don't know

How were non-study treatments defined in these questions? Check all that apply:

[ ] Don't know

[ ] By treatment class (e.g. painkillers)

[ ] By treatment name (e.g. paracetamol)

[ ] Other

Which of the following were explicitly asked about during *structured* questioning? Check all that apply:

[ ] Prescription medicines

[ ] Over the counter medicines

[ ] Traditional treatments

[ ] Supplements

[ ] Vaccinations

[ ] Other

How were options presented? Check all that apply:

[ ] Staff used the exact words as prepared

[ ] Staff re-phrased items in their own words

[ ] Items were only presented if they had been reported at a previous visit

[ ] Other

Please describe any non study-specific questions asked:

( ) Don't know

( ) Question(s) used: _________________

28) Were participants (or their caregiver) questioned about their *use of non-study treatments* in *another way* to the general or structured method, e.g. picture tool, diary?

( ) Yes

( ) No

( ) Don't know

Please briefly describe the method(s):

29) What was the rationale for using the above whole approach for questioning about *use of non-study treatments* in this study?

30) Does the above approach to questioning participants (or their caregivers) about their *use of non-study treatments* reflect normal practice in *most* malaria clinical studies you have been involved in?

( ) Yes

( ) No

( ) Other: _________________

Please briefly describe other questioning methods or combinations of methods used:

31) What do you consider as an *optimal* (important and feasible) approach for asking participants (or caregivers) about their *use of non-study treatments* to collect previous and concomitant medication data:

( ) The approach reported above

( ) A different approach to my above report (briefly describe): _________________

32) Why do you consider that optimal?

Please consider your *most recent malaria* clinical research study where participants (or their caregiver, such as a parent) were asked about adherence to treatment(s) given during the study, when answering the questions on this page.

33) What was the study drug regimen?

34) Did you collect individual participant data on drug intake?

( ) Yes

( ) No

( ) Don't know

35) What was the reason for not collecting such data?

( ) Don't know

( ) Reason(s): _________________

36) How did you collect the data? Check all that apply:

[ ] Dose observed

[ ] Participant (or caregiver) recall

[ ] Pill count (manual/electronic)

[ ] Dispensing confirmation

[ ] Pill diary

[ ] Other

Were all drug doses observed?

( ) All doses observed

( ) Some doses observed

( ) Don't know

37) Which data did you record? Check all that apply:

[ ] Quantity of dose dispensed

[ ] Dose given

[ ] Duration of total therapy

[ ] Time of dose

[ ] Whether participant vomited

[ ] Reason(s) for non-adherence

[ ] Whether taken with food/drink

[ ] Other

Was time of dose recorded for all drug doses?

( ) Time was recorded for all doses

( ) Time was recorded for some doses

( ) Don't know

When it was recorded that a participant vomited, what action was taken and how was this recorded?

38) How did you define adherence levels in your study?

39) Did you report adherence in the trial report?

( ) Yes

( ) No

( ) Don't know

General

41) We would appreciate if you would suggest any *references* that you know to be relevant for this work. Please describe any references you may know of here:

42) Would you like to hear about future projects for this topic?

[ ] Yes

Thank you for taking the time to complete this survey, we appreciate your input into this complex topic.
